# Association between metabolic syndrome and early-stage colorectal cancer

**DOI:** 10.1186/s12885-023-11537-3

**Published:** 2023-10-23

**Authors:** Chenchen Zhang, Liting Zhang, Yan Tian, Bingxin Guan, Shuai Li

**Affiliations:** 1 Department of Gastroenterology, the Second Hospital, Cheeloo College of Medicine, Shandong University, Beiyuan Street & 247, Jinan, Shandong 0531 China; 2 Department of Gastrointestinal Endoscopy Center, the Second Hospital, Cheeloo College of Medicine, Shandong University, Jinan, China; 3 Department of Pathology, the Second Hospital, Cheeloo College of Medicine, Shandong University, Jinan, China

**Keywords:** Metabolic syndrome, Early-stage colorectal cancer, Age, Serrated lesions

## Abstract

**Background:**

Accumulating studies have suggested metabolic syndrome (MetS) contributed to colorectal cancer (CRC) development. However, advanced CRC might decrease the detection proportion of MetS due to chronic malnutrition, we included patients with early-stage CRC to examine the associations among MetS, onset age, and different tumorigenesis pathways of CRC.

**Methods:**

We conducted a retrospective study that included 638 patients with early-stage CRC from January 2014 to December 2018. Patient information was collected from the medical record system and further refined during the follow-up. Stratified analyses of the associations between MetS and different stratification factors were determined by the Cochran‒Mantel‒Haenszel test.

**Results:**

There were 16 (13.3%) and 111 (21.4%) cases suffering from MetS in the early-onset and late-onset CRC groups, respectively. MetS coexisted in early-stage CRC patients ≥ 50 years of age more frequently than patients < 50 years of age (OR 1.77; 95% CI 1.01 to 3.12), but not for women patients (OR 0.84; 95% CI 0.79 to 0.90). MetS patients were associated with a higher risk of advanced serrated lesions than that of conventional adenomas (OR 1.585; 95% CI 1.02 to 2.45), especially in patients ≥ 50 years (OR 1.78; 95% CI 1.11 to 2.85).

**Conclusions:**

Metabolic dysregulation might partly contribute to the incidence of colorectal serrated lesions. Prevention of MetS should be highly appreciated in the early diagnosis and early treatment of the colorectal cancer system, especially in patients ≥ 50 years.

**Supplementary Information:**

The online version contains supplementary material available at 10.1186/s12885-023-11537-3.

## Introduction

Attributed to the screening efforts and removal of precancerous adenomas with colonoscopy nationwide, colorectal cancer (CRC) incidence and mortality rates tend to stabilize and decrease in highly developed countries [1, 2]. In contrast, the incidence of CRC in patients younger than 50 years (early-onset CRC) in the United States and other high-income countries is increasing alarmingly [2–4]. However, a worrying rise in both early-onset CRC and late-onset CRC (defined as cancer presenting in patients ≥ 50 years) has been observed over the past decade in China [5, 6]. Although geographic residence, genetic, ethnicity, and environmental factors might account for the incidence patterns, the exact reasons are not fully clarified. The globally increasing prevalence of metabolic syndrome (MetS) might play a part in the incidence of CRC [7–9].

MetS forms a constellation of metabolic dysregulations including insulin resistance, hypertension, central obesity, hypertriglyceridemia, and low high-density lipoprotein cholesterol (HDL-C) [8], and has become an epidemic condition affecting 25.6–39.9% of the population [10–12]. If left untreated, MetS directly impacts health and increases the risk of developing cancer [13–15]. Much evidence supports that MetS may contribute to the development and progression of CRC, specifically early-onset CRC [16–18]. Previous studies that enrolled patients with stage 0-IV CRC did not assess the associations between MetS and late-onset CRC in detail. In fact, patients with advanced CRC experience weight loss and low levels of blood glucose and lipids due to chronic malnutrition. Thus, the complicated associations between MetS and CRC need to be further explored. Early-stage CRC is defined as cancer cells that are confined to the mucosa and submucosa without metastasis to the regional lymph nodes or other organs [19]. It mainly included those lesions diagnosed as harbour high-grade dysplasia, in-situ carcinoma, or superficial submucosal invasion, which could be completely removed through curative colorectal endoscopic submucosal dissection (ESD)/endoscopic mucosal resection (EMR) [20]. Therefore, the 5-year survival rate is greater than 90%, and the prognosis is satisfactory. The majority of patients with early-stage CRC are not vulnerable to undernutrition which could impact existing metabolic dysregulations.

Insulin resistance and chronic inflammation might account for the association between MetS and CRC. A recent study showed that circadian disruption induced by imbalances in the gut microbiota negatively affects host metabolism, leading to MetS, which further activates oncoproteins and tumour growth [13]. Most CRCs originate from precancerous conventional adenomas and serrated lesions. Adenomatous tumorigenesis develops from continual DNA replication-induced mutations of self-renewing colorectal stem cells, while serrated tumorigenesis develops from the wound-healing process of colon epithelium caused by gut microbiota [21]. Thus, a small amount of information could help to clarify whether MetS is related to the two most common pathways of CRC development. To address these issues, we conducted this retrospective analysis enrolling patients with early-stage CRC to comprehensively examine the associations among MetS, onset age, and different tumorigenesis pathways of CRC.

## Materials and methods

### Study population

We performed a retrospective study at the Second Hospital, Cheeloo College of Medicine, Shandong University (Shandong, China) between January 2014 and December 2018. Patients older than 18 years who underwent curative colorectal ESD/EMR for the management of selected early-stage CRC were included. Exclusion criteria included coexisting other cancers or severe systemic diseases, a personal history of inflammatory bowel disease, Lynch syndrome, hereditary polyposis syndromes (familial adenomatous polyposis and hereditary nonpolyposis CRC), and undetailed medical history. This study was approved by the ethics committee of the Second Hospital, Cheeloo College of Medicine, Shandong University, and exempted from participants’ informed consent as all personal information had already been deidentified.

### Data collection and study definitions

Patients scheduled for colorectal ESD/EMR had preoperative testing ordered with selectivity, including a physical examination, laboratory tests, and CT scanning. Patient information, including demographics, laboratory reports, personal and family histories, colonoscopy results, and pathology reports, was collected from the medical record system, and further refined during the follow-up.

#### Body mass index (BMI)

Weight was measured to the nearest 0.1 kg using an electronic weighing scale with the patient removing shoes, and height to the nearest 1 cm using a folding stadiometer. BMI was calculated as weight in kilograms divided by the height squared in meters. BMI ranges of overweight were 24.0-27.9 kg/m^2^, and obesity ≥ 28.0 kg/m^2^ [10].

#### Blood pressure

Two blood pressure were measured > 60 s apart with an automated sphygmomanometer (OMRON HEM-7136, OMRON HEALTHCARE Co., LTD) after 5 min of meditation, and the average was analyzed in the study.

#### Serum biomarkers

Blood from the antecubital vein was obtained after ≥ 8 h of fasting. After centrifuged, serum was stored at − 70 °C until batch analysis at a central processing facility. Glucose was measured using the Glucose HK Gen.3 reagent (Roche Diagnostics GmbH), triglycerides using the Triglyceride GB reagent (Roche), and HDL-C using the HDL-Cholesterol Plus 3rd Generation Direct Method (Roche). All tests were performed on the Roche Modular P Chemistry analyzer (Roche Diagnostics Corporation).

#### MetS criteria

According to the diagnostic criteria recommended by the Chinese Diabetes Society, MetS constituted at least three of the following conditions: obesity/overweight, fasting blood glucose ≥ 6.1 mmol/L (110.0 mg/dL), or treatment for diabetes, systolic/diastolic blood pressure ≥ 130/85 mmHg or treatment for hypertension, and fasting triglycerides ≥ 1.7 mmol/L or HDL-C < 1.04 mmol/L.

Early-stage CRC was defined as an advanced colorectal neoplasia (a polyp with at least 25% villous features, high-grade dysplasia, or 10 mm or more in diameter) or carcinoma (mucosal cancer [T1a] or superficial submucosal cancer [T1b] with a depth of invasion less than 1000 μm below the muscularis mucosae). Additionally, advanced colorectal neoplasia was histologically categorized into two common subtypes: conventional adenomas consisting of tubular adenomas and tubulovillous adenomas, and serrated lesions consisting of sessile serrated lesions and traditional serrated adenomas. Patients with multiple early-stage CRC were classified according to the neoplasia with higher pathological grade. Each pathological report was reviewed by an experienced pathologist to avoid interobserver bias. Histopathological diagnoses were based on the 2019 WHO classification of tumour of the digestive system [22]. Current or past smokers were defined as those who smoked ≥ 7 cigarettes each week and current or past drinkers were defined as those who drink ≥ 2 times each week. Oral administration of aspirin or metformin ≥ 2 times per week over 1 year was defined as regular intake of aspirin/metformin [23].

### Statistical analysis

The count data are displayed as numbers and percentages and describe the basic features of the population enrolled, clinicopathological features of early-stage CRC, and metabolic comorbid conditions of two age cohorts, which were compared using chi-square tests. The Bonferroni correction method was used for multiple testing. We evaluated the association between MetS and the onset age of early-stage CRC as the main analysis. In addition, we examined the relationship between MetS and the two most common pathways of CRC development. As secondary analyses, we investigated whether the identified associations differ according to sex, age, family history, smoking status, alcohol consumption, and intake of aspirin. Stratified analyses of the associations between MetS and different stratification factors were determined by the Cochran‒Mantel‒Haenszel test. P for interaction was calculated by Breslow-Day test of homogeneity using the cross-product terms of MetS and each stratification factor. Odds ratios (ORs) and 95% confidence intervals (CI) for each factor were calculated to assess impact capacity. Finally, we employed a propensity score analysis to further avoid confounding bias, and match tolerance was set as 0.02. Statistical analyses were performed using the SPSS (version 25.0; IBM Corp, Armonk, NY) and MedClac version 20.0 (MedCalc Software Ltd, Ostend, Belgium), and statistical significance was set at 0.05.

## Results

### Baseline features of patients and early-stage CRC included in this study

A total of 120 early-onset CRC cases and 518 late-onset CRC cases were recruited in our analyses, and the average age of diagnoses was 43.5 ± 4.5, and 63.2 ± 8.3, respectively. All patients underwent colonoscopy and CT scanning 1, 2, 3, and 5 years after the initial treatment. No local tumor recurrence or distal metastasis was discovered during a 3-year follow-up. The detailed characteristics of the two cohorts were listed in Table 1, which were similar according to smoking, alcohol consumption, intake of aspirin and metformin, and history of FDR with CRC. Compared with the early-onset CRC cohort, the proportions of women and carcinoma in patients ≥ 50 years of age were higher (P *<* 0.05).

**Table 1:** Baseline features of patients included in this study

	Early-onset CRC cohort (N = 120)	Late-onset CRC cohort (N = 518)	P
Age (years), mean ± SD	43.5 ± 4.5	63.2 ± 8.3	
Sex, Men, n (%)	91 (75.8)	327 (63.1)	**0.008**
Smoking, n (%)	37 (30.8)	168 (32.4)	0.735
Alcohol consumption, n (%)	55 (45.8)	224 (43.2)	0.606
Intake of aspirin, n (%)	9 (7.5)	73 (14.1)	0.052
Intake of metformin, n (%)	6 (5.0)	48 (9.3)	0.130
History of FDR with CRC, n (%)			0.707
None	116 (96.7)	504 (97.3)	
One or more	4 (3.3)	75 (2.7)	
Patients with colorectal neoplasia, n (%)			**0.016**
Advanced colorectal neoplasia	113 (94.2)	446 (86.1)	
Carcinoma	7 (5.8)	72 (13.9)	

Significant P values are shown in bold text

Abbreviations: CRC, colorectal cancer; FDR, first-degree relative

The clinicopathological features of early-stage CRC resected across this study were displayed in Table 2 and were similar in the two cohorts stratified by size and location. It should be noted that the early-onset CRC cohort was more likely to have conventional adenomas other than serrated lesions. The multiple comparison analysis results were provided in the Table S1.

**Table 2:** Clinicopathological features of early-stage CRC.

	Early-onset CRC cohort (N = 120), n (%)	Late-onset CRC cohort (N = 518), n (%)	P
Pathology			**0.037**
Carcinoma	7 (5.8)	72 (13.9)	
Conventional adenomas	85 (70.8)	318 (61.4)	
Tubular	75 (62.5)	268 (51.7)	
Tubulovillous	10 (8.3)	50 (9.7)	
Serrated lesions	28 (23.3)	128(24.7)	
Sessile serrated lesions	19 (15.8)	68 (13.1)	
Traditional serrated adenoma	9 (7.5)	60(11.6)	
Size			0.918
< 10 mm	19 (15.8)	84 (16.2)	
≥ 10 mm	101 (84.2)	434 (83.8)	
Location			0.170
Proximal colon	45 (37.5)	225 (43.4)	
Distal colon	61 (50.8)	215 (41.5)	
Rectal	14 (11.7)	78 (15.1)	

Significant P values are shown in bold text

Abbreviations: CRC, colorectal cancer

### Comparison of metabolic comorbid conditions in two age cohorts

**Fig. 1:**
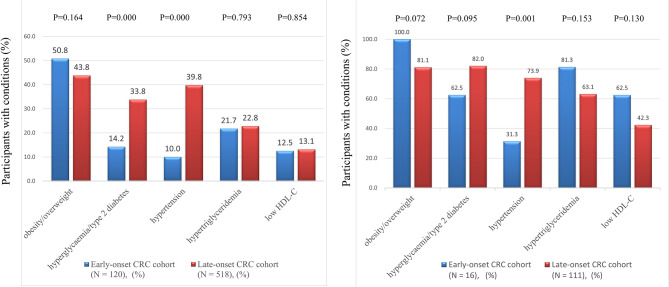
Comparison of metabolic comorbid conditions in two age cohorts. (**a**) early-stage CRC patients (N = 638); (**b**) early-stage CRC patients complicated with metabolic syndrome (N = 127). Abbreviations: CRC, colorectal cancer; HDL-C, high-density lipoprotein cholesterol

In the early-onset CRC cohort, 17 (14.2%) patients were complicated with hyperglycemia/type 2 diabetes, and 12 (10.0%) patients had hypertension. In contrast, the incidence of hyperglycemia/type 2 diabetes and hypertension in the late-onset CRC cohort was 33.8% and 39.8%, respectively, which was significantly higher than that of the former (Fig. 1a).

Among metabolic comorbid conditions in patients < 50 years with MetS, obesity/overweight (100.0%) and hypertriglyceridaemia (81.3%) accounted for the highest proportion. In patients ≥ 50 years with MetS, obesity/overweight (81.1%) and hyperglycemia/type 2 diabetes (82.0%) were the most common comorbid conditions (Fig. 1b).

### The association between MetS and two age cohorts

**Fig. 2:**
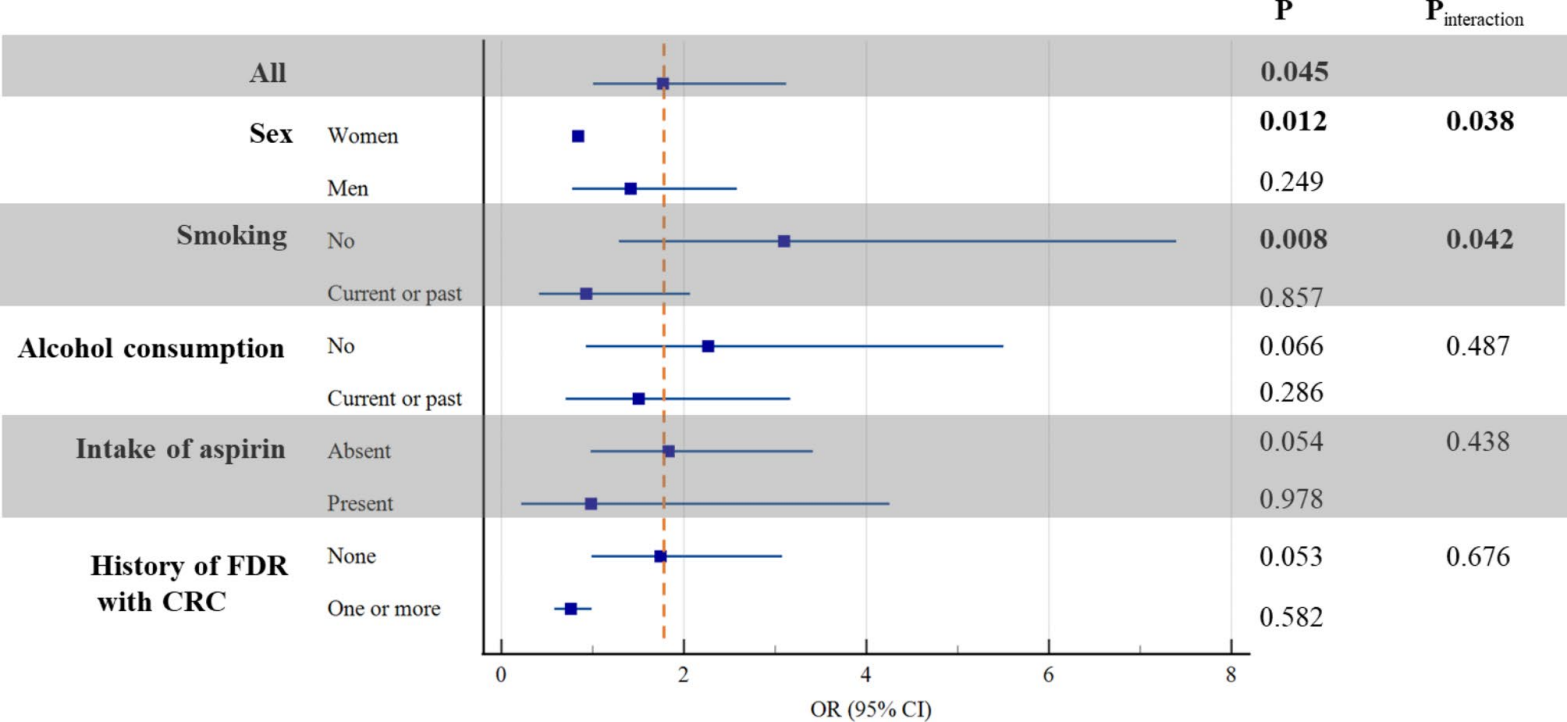
Stratified analyses for metabolic syndrome and two age cohorts (late-onset CRC cohort/early-onset CRC cohort). The concrete data of each covariate was shown in Table 3. Abbreviations: CRC, colorectal cancer; FDR, first-degree relative

There were 16 (13.3%) and 111 (21.4%) cases suffering from MetS in the early-onset and late-onset CRC groups, respectively (Table 3). As shown in Fig. 2, MetS coexisted in early-stage CRC patients ≥ 50 years of age more frequently than patients < 50 years of age (OR 1.77; 95% CI 1.01 to 3.12), but not for women patients (OR 0.84; 95% CI 0.79 to 0.90). The positive association between MetS and onset age of early-stage CRC patients was similar when restricted to alcohol consumption, intake of aspirin, and history of FDR with CRC (all P_interaction_ > 0.05), while the relationship appeared stronger for individuals with no smoking (OR 3.10; 95% CI 1.29 to 7.40). When comparing the two cohorts through the propensity score analysis (more details were shown in Table S2), 42.5% of the late-onset CRC had MetS among participants over age 50 compared with only 13.3% of the controls (P = 0.000).

**Table 3:** Stratified analyses of the association between MetS and two age cohorts

Stratification		MetS	Early-onset CRC cohort (N = 120), n (%)	Late-onset CRC cohort (N = 518), n (%)
All		without	104 (86.7)	407 (78.6)
		with	16 (13.3)	111 (21.4)
Sex	Women	without	29 (24.2)	156 (30.1)
		with	0 (0)	35 (6.8)
	Men	without	75 (62.5)	251 (48.5)
		with	16 (13.3)	76 (14.7)
Smoking	No	without	77 (64.2)	282 (54.4)
		with	6 (5.0)	68 (13.1)
	Current or past	without	27 (22.5)	125 (24.1)
		with	10 (8.3)	43 (8.3)
Alcohol consumption	No	without	59 (49.2)	239 (46.1)
		with	6 (5.0)	55 (10.6)
	Current or past	without	45 (37.5)	168 (32.4)
		with	10 (8.3)	56 (10.8)
Intake of aspirin	Absent	without	98 (81.7)	358 (69.1)
		with	13 (10.8)	87 (16.8)
	Present	without	6 (5.0)	49 (9.5)
		with	3 (2.5)	24 (4.6)
History of FDR with CRC	Absent	without	100 (83.3)	394 (76.1)
		with	16 (13.3)	110 (21.2)
	Present	without	4 (3.3)	13 (2.5)
		with	0 (0)	1 (0.2)

Abbreviations: CRC, colorectal cancer; FDR, first-degree relative; MetS, metabolic syndrome

### The relationship between MetS and two common pathological classifications of advanced colorectal neoplasia

**Fig. 3:**
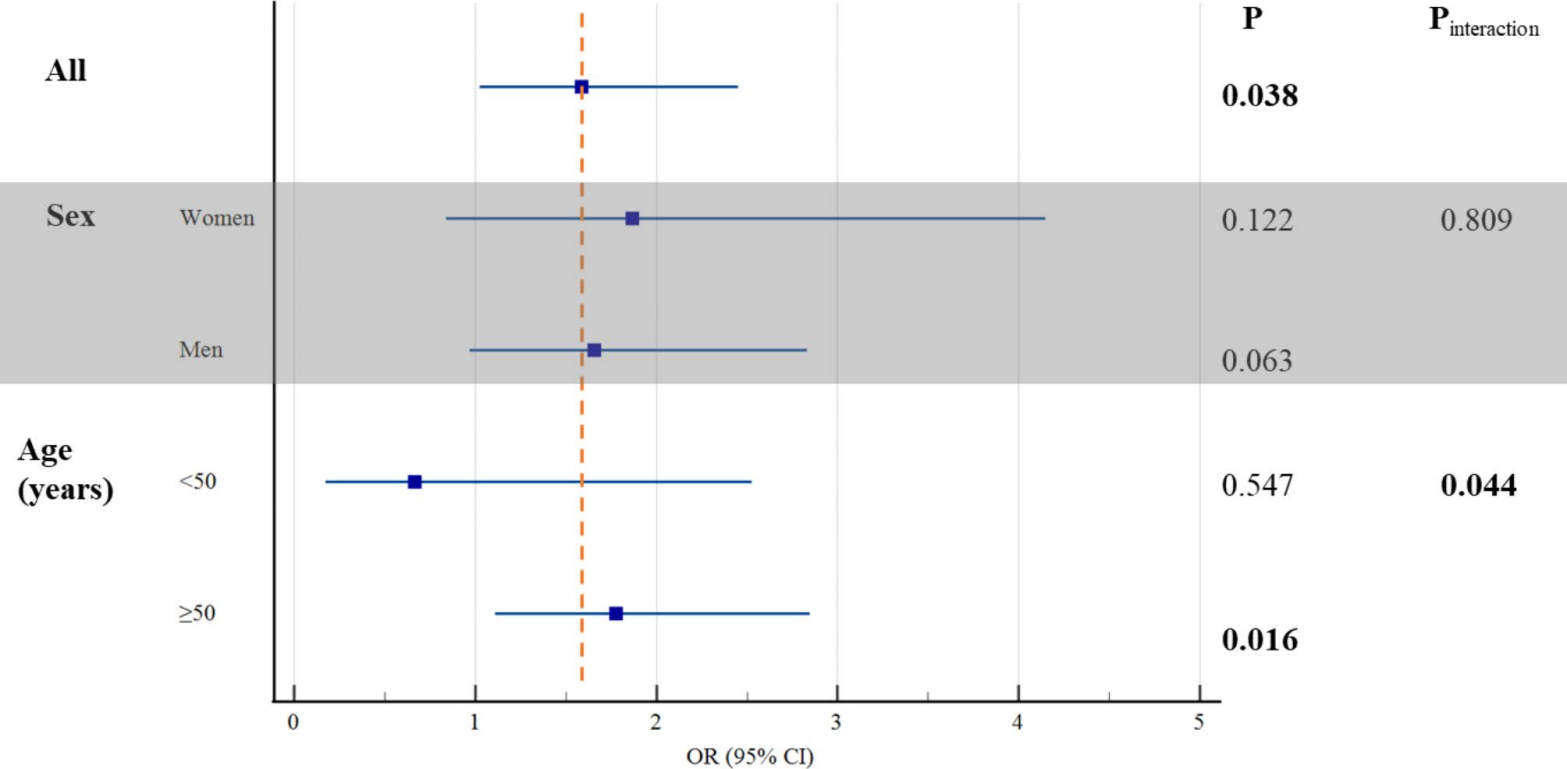
Stratified analyses of the association between metabolic syndrome and two common pathological classifications of advanced colorectal neoplasia (serrated lesions group/ conventional adenomas group). The concrete data of each covariate was shown in Table 4

The early-stage CRC patients enrolled without carcinoma were classified into two groups according to the pathology of the neoplasia resected through EMR or ESD: conventional adenomas group and serrated lesions group. The basic information of the two groups was listed in Table 4. MetS patients were associated with a higher risk of advanced serrated lesions than that of conventional adenomas (OR 1.585; 95% CI 1.02 to 2.45), and the propensity score analysis came to the same result (26.3% vs. 13.5%, P = 0.005; more details were shown in Table S3). Furthermore, we assessed this association according to sex and age (Fig. 3). Generally, the association between MetS and advanced serrated lesions was more statistically significant for increasing age (OR 1.78; 95% CI 1.11 to 2.85).

**Table 4:** Stratified analyses of the association between MetS and two common pathological classifications of advanced colorectal neoplasia

Stratification		MetS	Conventional adenomas group (N = 403), n (%)	Serrated lesions group (N = 156), n (%)
All		without	329 (81.6)	115 (73.7)
		with	74 (18.4)	41 (26.3)
Sex	Women	without	108 (26.8)	54 (34.6)
		with	15 (3.7)	14 (9.0)
	Men	without	221 (54.8)	61 (39.1)
		with	59 (14.6)	27 (17.3)
Age (years)	< 50	without	72 (17.9)	25 (16.0)
		with	13 (3.2)	3 (1.9)
	≥ 50	without	257 (63.8)	90 (57.7)
		with	61 (15.1)	38 (24.4)

Note: Advanced colorectal neoplasia was defined as a polyp with at least 25% villous features, high-grade dysplasia (HGD), or 10 mm or more in diameter

Abbreviations: MetS, metabolic syndrome

## Discussion

Accumulating evidence indicates that MetS might contribute to the incidence of CRC [7, 9, 15, 17, 24–26]. A recent nested case–control study discovered MetS was related to increased risk of CRC diagnosed at age 18–49 (OR 1.25; 95% CI 1.09 to 1.43) and age 50–64 (OR 1.21; 95% CI 1.15 to 1.27) [16]. Leveraging real-world inpatient data that covers 638 patients with early-stage CRC who were 31–92 years of age, we found that MetS coexisted in early-stage CRC patients over 50 years of age more frequently than patients < 50 years of age and this result was still significant after adjusting for potential risk factors for CRC. We also found that MetS was associated with an increased risk of advanced serrated lesions compared with conventional adenomas, and this positive association was strongly driven by increasing age.

Ageing is characterized by a gradual decline of physiological function over time. Research in epidemiology has shown that the incidence of MetS increases with age [11]. Not surprisingly, the ageing process and MetS have many biochemical changes in common. Waist circumstance, the most widely used measurement for central obesity, increases with age and other metabolic comorbid conditions [27]. Furthermore, among the essential mechanisms in the progression of MetS, insulin resistance, chronic inflammation, and neurohormonal activation also greatly plague elderly patients [8, 28]. In addition, various therapeutic efforts to improve insulin resistance, hypertriglyceridaemia, and central visceral obesity associated with the MetS, for example, physical activity [29], Mediterranean diet [30], Butyrate [31], metformin and stains [32], have been verified to be safe and effective for patients ≥ 50 years. Additionally, parts of treatment protocols could prevent the development and metastasis of CRC [2, 33]. We observed that late-onset CRC patients harbour more metabolic comorbid conditions and a stronger association with MetS than early-onset CRC patients. This finding was similar to that of a previous study that reported a higher colorectal adenoma risk in MetS patients over 50 years of age [17]. Another study on the relationship between MetS and CRC showed that CRC patients complicated with MetS are diagnosed at an advanced age, mostly over 60 years [34]. Interestingly, when stratified by sex in our study, the positive association between MetS and onset age of early-stage CRC was no longer apparent for women. Collectively, our findings offer preliminary support for a major part of metabolic dysregulation in late-onset CRC. MetS patients over 50 years of age should be a risk population for CRC screening and could benefit from therapeutic efforts against MetS to prevent the development of CRC.

Different from conventional adenomas arising from mutant stem cells in a ‘‘bottom-up’’ fashion, serrated polyps might originate from metaplasia of differentiated cells at the colon luminal surface through a ‘‘top-down’’ fashion. Altered microbiota have been associated with the tumorigenesis of serrated lesions and particular microbial species might drive the epithelial damage-induced metaplastic process as the “first hit” [21, 35, 36]. Many studies have revealed that the gut microbiota can participate in the development of insulin resistance [37, 38]. Another study considered bacterial lipopolysaccharide from gut microbiota as the trigger of metabolic endotoxaemia related to MetS [39]. Additionally, studies have shown that MetS is associated with intestinal microbiota disorders, such as a lower proportion of Bacteroidetes/Firmicutes, an increased abundance of Proteobacteria, and alterations of Akkermansia and Bifidobacteriaceae [40–43]. One of the possible mechanisms for the association between MetS and microbiota dysbiosis was circadian disruption, which could negatively affect healthy metabolic function through dynamic crosstalk with gut microbiota [13]. In this study, we also examined the association between MetS and two common pathological classifications of advanced colorectal neoplasia and reported a positive association between MetS and advanced serrated lesions, especially in the elderly population. The reasons behind these findings remain unclear, as few studies have examined such issues. Future investigations based on microbiota dysbiosis and the cytotoxic microenvironment would be critical in clarifying the mechanisms linking MetS and serrated lesions.

There were several strengths in our study. First, the fasting serum biomarkers and measured anthropometric variables of all patients enrolled were available from the medical record system. Second, advanced CRC might decrease the detection proportion of concomitant comorbid conditions; therefore, we included patients with early-stage CRC to reduce detection bias. Third, we adjusted for a few covariates associated with risk factors for CRC such as smoking, alcohol consumption, intake of aspirin, and history of FDR with CRC, and the stratified analyses also provided effective support for the credibility of the results. Furthermore, we used BMI as the alternative to waist circumstance for the diagnostic index of central obesity, which might result in misestimating the prevalence of MetS. Moreover, the single-centre analysis including a small sample size in a relatively homogenous population could limit the adaptability of the results in real-world situations. Finally, information about other risk factors such as sedentary lifestyle and red or processed meat intake was not available, both of which were related to MetS and CRC in the elderly population. However, these factors are relatively moderate risk [2, 9], and the potential influence of the confounding was considered modest.

## Conclusions

This study showed that metabolic dysregulation might partly contribute to the incidence of colorectal serrated lesions, and additional studies on the mechanisms are warranted. Given the increasing prevalence of MetS at the population level, our analysis suggested that prevention of MetS should be highly appreciated in the early diagnosis and early treatment of the colorectal cancer system, especially in patients ≥ 50 years.

### Electronic supplementary material

Below is the link to the electronic supplementary material.

Supplementary Material 1

Supplementary Material 2

Supplementary Material 3

## Data Availability

The datasets used during the current study are available from the corresponding author upon reasonable request.

## Abbreviations

BMI: body mass index
CI: confidence interval
CRC: colorectal cancer
EMR: endoscopic mucosal resection
ESD: endoscopic submucosal dissection
FDR: first-degree relatives
HDL-C: high-density lipoprotein cholesterol
HGD: high-grade dysplasia
MetS: metabolic syndrome
OR: odds ratio
