# Supplementary material for: Association between metabolic syndrome and early-stage colorectal cancer
Source: BMC Cancer. 2023 Oct 23;23:1020. doi: 10.1186/s12885-023-11537-3 (PMC10591414; doi:10.1186/s12885-023-11537-3)
Supplement: Supplementary file 1 — Supplementary Material 1 [file 12885_2023_11537_MOESM1_ESM.docx]

Table S1. The pathological and location features of early-stage CRC resected across this study.

|  | Early-onset CRC cohort  (N = 120), n (%) | Late-onset CRC cohort  (N = 518), n (%) | P |
| --- | --- | --- | --- |
| Pathology |  |  |  |
| Carcinoma | 7 (5.8) | 72 (13.9) | reference |
| Conventional adenomas | 85 (70.8) | 318 (61.4) | > 0.05 |
| Serrated lesions | 28 (23.3) | 128(24.7) | < 0.05 |
| Location |  |  |  |
| Proximal colon | 45 (37.5) | 225 (43.4) | reference |
| Distal colon | 61 (50.8) | 215 (41.5) | > 0.05 |
| Rectal | 14 (11.7) | 78 (15.1) | > 0.05 |

Bonferroni correction was used for multiple tests.

Abbreviations: CRC, colorectal cancer.
