# Supplementary material for: Association between metabolic syndrome and early-stage colorectal cancer
Source: BMC Cancer. 2023 Oct 23;23:1020. doi: 10.1186/s12885-023-11537-3 (PMC10591414; doi:10.1186/s12885-023-11537-3)
Supplement: Supplementary file 2 — Supplementary Material 2 [file 12885_2023_11537_MOESM2_ESM.docx]

Table S2. Propensity score matching of association between MetS and two age cohorts

| Stratification | | Mets | Early-onset CRC cohort  (N = 120), n (%) | Late-onset CRC  cohort  (N = 120), n (%) |
| --- | --- | --- | --- | --- |
| All | | without | 104 (86.7) | 69 (57.5) |
|  |  | with | 16 (13.3) | 51 (42.5) |
| Sex | Women | without | 29 (24.2) | 13 (10.8) |
|  |  | with | 0 (0) | 4 (3.3) |
|  | Men | without | 75 (62.5) | 56 (46.7) |
|  |  | with | 16 (13.3) | 47 (39.2) |
| Smoking | No | without | 77 (64.2) | 58 (48.3) |
|  |  | with | 6 (5.0) | 27 (22.5) |
|  | Current or past | without | 27 (22.5) | 11 (9.2) |
|  |  | with | 10 (8.3) | 24 (20.0) |
| Alcohol consumption | No | without | 59 (49.2) | 33 (27.5) |
|  |  | with | 6 (5.0) | 17 (14.2) |
|  | Current or past | without | 45 (37.5) | 36 (30.0) |
|  |  | with | 10 (8.3) | 34 (28.3) |
| Intake of aspirin | Absent | without | 98 (81.7) | 69 (57.5) |
|  |  | with | 13 (10.8) | 51 (42.5) |
|  | Present | without | 6 (5.0) | 0 (0.0) |
|  |  | with | 3 (2.5) | 0 (0.0) |
| History of FDR with CRC | Absent | without | 100 (83.3) | 62 (51.7) |
|  |  | with | 16 (13.3) | 50 (41.7) |
|  | Present | without | 4 (3.3) | 7 (5.8) |
|  |  | with | 0 (0) | 1 (0.8) |

Abbreviations: CRC, colorectal cancer; FDR, first-degree relative; MetS, metabolic syndrome.
