# Supplementary material for: Association between metabolic syndrome and early-stage colorectal cancer
Source: BMC Cancer. 2023 Oct 23;23:1020. doi: 10.1186/s12885-023-11537-3 (PMC10591414; doi:10.1186/s12885-023-11537-3)
Supplement: Supplementary file 3 — Supplementary Material 3 [file 12885_2023_11537_MOESM3_ESM.docx]

Table S3. Propensity score matching of association between MetS and two common pathological classifications of advanced colorectal neoplasia

| Stratification | | MetS | Conventional adenomas group  (N = 156), n (%) | Serrated lesions  group  (N = 156), n (%) |
| --- | --- | --- | --- | --- |
| All | | without | 135 (86.5) | 115 (73.7) |
|  |  | with | 21 (13.5) | 41 (26.3) |
| Sex | Women | without | 47 (30.1) | 54 (34.6) |
|  |  | with | 8 (5.1) | 14 (9.0) |
|  | Men | without | 88 (56.4) | 61 (39.1) |
|  |  | with | 13 (8.3) | 27 (17.3) |
| Age (years) | < 50 | without | 31 (19.9) | 25 (16.0) |
|  |  | with | 5 (3.2) | 3 (1.9) |
|  | ≥ 50 | without | 104 (66.7) | 90 (57.7) |
|  |  | with | 16 (10.3) | 38 (24.4) |

Note: Advanced colorectal neoplasia was defined as a polyp with at least 25% villous features, high-grade dysplasia (HGD), or 10 mm or more in diameter.

Abbreviations: MetS, metabolic syndrome.
